# Environmental Complexity: Additional Human Visual Contact Reduced Meat Chickens’ Fear of Humans and Physical Items Altered Pecking Behavior

**DOI:** 10.3390/ani12030310

**Published:** 2022-01-27

**Authors:** Peta S. Taylor, Paul H. Hemsworth, Jean-Loup Rault

**Affiliations:** 1 School of Environmental and Rural Science, University of New England, Armidale, NSW 2350, Australia; 2 Animal Welfare Science Centre, University of Melbourne, Parkville, VIC 3052, Australia; phh@unimelb.edu.au; 3 Institute of Animal Welfare Science, University of Veterinary Medicine Vienna, Veterinaerplatz 1, 1210 Vienna, Austria; Jean-Loup.Rault@vetmeduni.ac.at

**Keywords:** enrichment, broiler, behavior, welfare, human-animal interaction, physiological stress

## Abstract

**Simple Summary:**

Environmental complexity can improve chicken welfare. However, the outcome of such environmental changes will depend on the resources provided. We provided either various physical items that posed no biosecurity risk and were inexpensive (such as balls, chains, a perch and rope) to alter behavioral time budgets and interactions with the environment or provided additional visual contact with humans (10 min daily) with the aim to reduce fearfulness. Additional human contact reduced fear of humans at 35 days of age, but did not affect general fearfulness. Increased environmental complexity via the provision of physical items reduced some indicators of fear at 21 days, but not at 35 days of age. A woodblock and perch were the most favored physical items, but chickens preferred to sit underneath the perch rather than on top. When pecking items were not provided, chickens redirected their pecking to the wood shaving litter. Overall, there was little evidence that our physical items improved the chickens’ behavioral time budget, fear, physiological stress or production. The benefits of additional visual contact with humans should be investigated on larger groups to ensure that such effects are practical and effective to reduce fear of humans on farm.

**Abstract:**

Increased environmental complexity can improve animal welfare, depending on the resources provided and use by the animal. We provided chickens either with physical items that posed no biosecurity risk and were inexpensive (balls, chains, perches and rope) (P; *n* = 36) or additional visual human contact (10 min daily) (HC; *n* = 36) compared to farm-like standard control groups (C; *n* = 36) with 3 pens per treatment. Additional human contact reduced fear of humans at 35 days of age, but not general fearfulness. P birds required more inductions to induce tonic immobility compared to HC and C birds at 21 days of age. However, other indicators of fear (open field test and plasma corticosterone concentration) did not significantly differ. P birds favored the woodblock for resting, and the perch but preferred to sit underneath the perch rather than on top. When pecking items were not provided, C and HC chickens redirected their pecking behavior toward the litter. Overall, there was little evidence that our physical items improved the chickens’ behavioral time budget, fear, physiological stress or production. Additional human contact should be investigated in large scale experiments to ensure its effectiveness to reduce fear of humans on farm.

## 1. Introduction

Environmental enrichment has the potential to improve captive animal welfare [1,2,3,4]. However, despite good intentions, not all proposed enrichment programs are effective and in fact, some have shown to negatively impact welfare, such as increasing fearfulness or mortality [5,6]. Some authors suggest that environmental enrichment devices must be biologically relevant to improve animal welfare [7,8], however there is evidence that increased environmental complexity (regardless of biological relevance) can improve welfare, for example by increasing stress resilience and reducing fearfulness [9,10]. 

Increasing complexity in an animal’s environment may improve agency which promotes learning and development, enhances skills for the future and improves competency when dealing with challenges later in life [11,12,13]. Such competency may be beneficial, or perhaps necessary, for challenges during the production cycle of meat chickens, such as access to an outdoor range (typically provided 21 days of age for fast-growing meat chickens in Australia) and handling and transportation during pick up and slaughter (typically between 35 and 45 days of age for fast-growing meat chickens in Australia). Furthermore, providing complex environments may also provide immediate welfare benefits by satisfying an animal’s intrinsic motivation to explore and acquire knowledge [11]. Appeasing intrinsic propensities for knowledge acquisition is likely associated with positive affect regardless of the functional outcome [14]. Evidence to support the feeling of reward associated with knowledge acquisition is demonstrated by contra-freeloading (i.e., chickens will work (forage) for food even when it is freely available) [15,16]. Supporting this theory, is recent research that has shown that environmental complexity provided to meat chickens resulted in improvements to positive affect [17]. Chickens that were provided with sand, pecking stones, perches and novel items that were not biologically relevant were more optimistic than chickens housed in standard conditions, suggesting environmental complexity improved the chicken’s mood [17]. However, the items provided to increase the complexity in an environment may be more important than the complexity of the environment per se. For example, rats that were provided with a new enrichment item each week (i.e., novelty) had poorer welfare than rats that were provided with all of the items at the same time (i.e., environmental complexity), evident by improved body and thymus weights, a reduction in aggression and other behavioral changes associated with positive affect such as increased rest and interaction with the environment [18]. However, an order effect was observed when the novel enrichments were provided, suggesting that some enrichments were more valued than others and the improvements to welfare from the environmental complexity group (i.e., all items provided at the same time) may have been associated with one (or more) enrichments alone rather than due to the complexity of the environment. Monitoring interactions with specific items provided to animals in complex environments may provide insight into what is truly enriching and valued. 

Additional complexity may be provided by stockpersons in commercial production systems, although the welfare outcome from this type of enrichment is dependent on the nature of the human interaction [19]. Positive handling consisting of human behaviors that avoid startling and encouraging approach has shown to reduce fear in meat chickens [20] and laying hens [21]. Positive human contact twice daily during the first six weeks of life reduced fear responses toward humans but more importantly (arguably) human contact also reduced physiological stress responses when birds were contained in a crate for 12 min [20]. The associated reduction to fearfulness when challenged by a non-human specific stimulus in the Hemsworth, Coleman, Barnett and Jones [20] study shows the potential of positive human-animal interactions to improve stress resilience and reduce general fearfulness (i.e., not stimulus-specific responses). Clearly, positive handling of each individual chickens each day, in flocks of often thousands of individuals, is impractical to implement. Zulkifli et al. [22] provides evidence that visual contact alone may have similar benefits to chicken welfare and would be much more practical to implement. However, Jones [21] found no impact of visual contact with humans on indicators of fearfulness. Yet Jones [21] did find shorter TI durations (indicative of reduced general fearfulness) of chickens if they observed their neighbor receiving additional positive human contact compared to chickens that had not received, or observed others receiving, additional positive contact and chickens that received negative human interaction (suspension by the legs). 

Reducing fearfulness in meat chickens is beneficial for chicken welfare, as chronic states of fear, anxiety and stress are associated with negative affect and disrupted biological functioning, and short-term exaggerated fear responses can lead to injuries and smothering. Additionally, reducing fear responses can positively impact production [20]. Hemsworth, Coleman, Barnett and Jones [20] showed that human avoidance (indicative of fear of humans) behavior in 22 commercial meat chicken flocks was associated with a reduction in feed conversion ratio (FCR) and the distance from the humans during a human avoidance test (HAV) predicted 28% of the FCR variance. Thus, reducing fearfulness of meat chickens can have benefits for the meat chickens themselves and the chicken meat industry. 

To further understand the effect of environmental complexity and positive human contact on meat chicken behavior and welfare, we provided meat chickens with either physical items or twice daily visual human contact. Specifically, our focus was to reduce fearfulness, physiological stress and increase exploration and agency. Thus, we hypothesized that meat chickens raised in more complex environments would interact with their home pen environment more frequently, approach a novel object faster and have lower general fearfulness than chickens raised in standard housing.

## 2. Materials and Methods

Day-old mixed-sex Ross308 chickens (*n* = 108; 55% female, 45% male) were allocated equally to either a control (C), additional environmental complexity with physical items (P) or additional environmental complexity with human contact (HC) group. They were housed in nine groups of 12 birds in 1 m^2^ pens, across three rooms. Each room contained one pen of each treatment, resulting in three pen replicates per treatment. All chickens were visually isolated from the other pens. The pen environment contained one drinker and feeder, wood shaving flooring and received human visual contact for approximately 10 min daily for daily care. Heat lamps were provided from day one until day five and therefore lighting was continuous for the first five days, then continued on a 16:8 L:D schedule. Temperature averaged 26 °C in week one and decreased overtime to 18 °C in week six. Commercial starter feed was provided from day one, grower feed from 14 days of age and finisher diet from 26 to 35 days of age (Barastoc, Ridley Agriproducts, Melbourne, VIC, Australia). From week three, litter was changed once a week with minimal interaction with the birds. 

**Figure 1:**
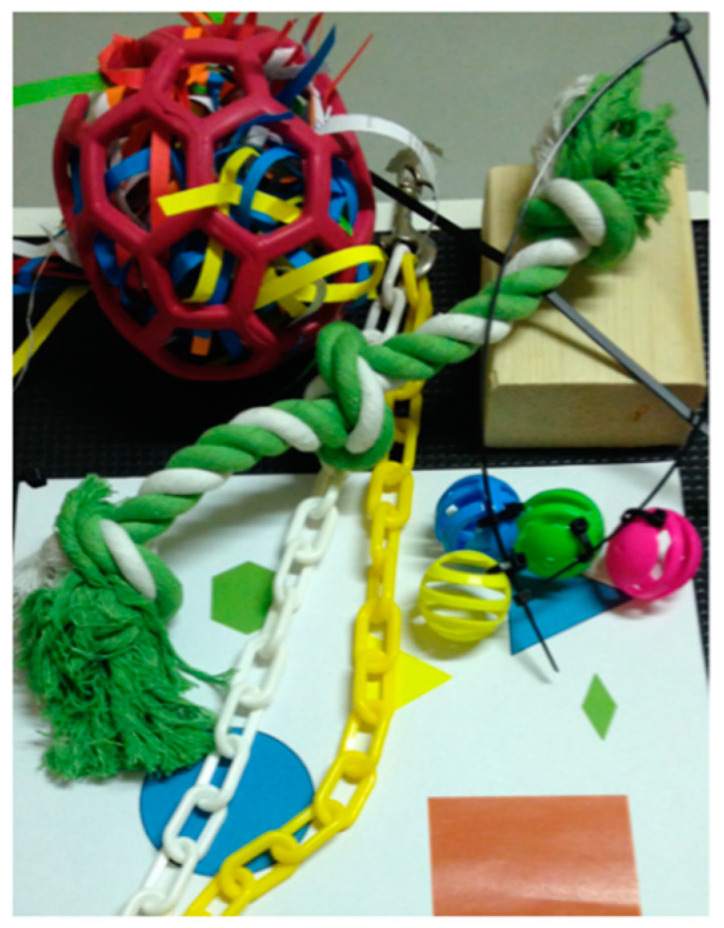
Environmental enrichment items provided to chickens in the environmental complexity with physical items (P) treatment group.

The P and HC treatments were applied from five days of age and were given until the end of the experiment at 35 days of age. Birds in the P treatment group were provided with additional physical items including a perch (non-treated pine; L 800 mm × W 70 mm × H 200 mm), a green and white cotton rope toy (500 mm L), yellow and white plastic chains (650 mm long), plastic balls (30 mm diameter, pink, green, blue and yellow attached loosely to a cable tie), shredded paper contained in a rubber ball (190 mm diameter, with multiple openings for chickens to pull out paper), a colored drawing (A3) and a small woodblock (untreated pine; L 25 mm × W 70 mm × H 200 mm). All moveable items were secured to a designated area of the pen via a cable tie. The location of each enrichment in the pen was randomly chosen but consistent between all P pens (Figure 1).

Birds in the HC treatment group were exposed twice daily (8.00 and 15.00 h) to a human for 5 min. Within the 5-min treatment period the human moved between each of the corners of the pen with three intermittent stationary periods (1 min each), in addition to routine human contact. No attempt to interact with birds was made by the human during the HC treatment however chickens could approach the human and interact (e.g., pecking at shoes). Two experimenters provided the human contact over the course of the experiment. Both experimenters wore the same white laboratory coat and wore dark colored pants and black boots.

Individual birds were randomly selected and either tested at 21–23 days or 35–37 days of age (*n* = 6/pen/age) for their fear responses via tonic immobility (TI), open field (OFT) and novel object (NOT) tests, in this order over three consecutive days (one test per day). Individual birds were identified via a Jiffy wing band (Bellsouth, Hastings, Victoria, Australia) attached during the first day of life and individuals tested within the same age group were marked with a colored spray (FIL Tell Tail, GAE, Mount Maunganui, New Zealand) to help identification during testing. Fear responses specific to a human stimulus was assessed at day 38 in a human approach (HAP) and human avoidance (HAV) test. A description of each test is outlined below. White laboratory coats were worn by all humans during daily care, implementing the HC treatment and during all behavioral tests.

### 2.1. Tonic Immobility

We utilized the tonic immobility method reported by Jones [21]. Each chicken was tested in a quiet isolated room, transported by one handler using two hands. The chicken was inverted and restrained gently on its back in a U-shaped cradle, the head was lightly covered and light pressure was applied to the sternum for 15 s. After 15 s of pressure, the handler removed the tactile interaction and moved away out of sight of the chicken until the chicken righted itself. A maximum of five attempts were made to induce the TI state. A successful induction was considered when the chicken remained in TI for more than 15 s after the handler released pressure. Chickens were permitted to remain in a TI state for a maximum of 600 s after which they were gently righted. If TI was not induced after five attempts, that chicken was given a score of zero. The bird’s response was captured by video recorders (Hero3, GoPro Inc., San Mateo, CA, USA) and the length of time that the chickens remained in TI was assessed from the recordings at a later time by one assessor who was blind to treatment. 

### 2.2. Open Field Test

Chickens were transported by hand to the test arena (1900 L × 920 W × 570 H) in a testing room < 5 m from their home pen but visually and audibly isolated. The testing room was identical to the rooms that contained the home pens and wood shaving litter flooring was provided in the test area, similar to the home pen environment. Chickens were placed in the center of the front third of the arena and left for five minutes. Behavior was recorded via GoPro digital camera (Hero3, GoPro Inc., San Mateo, CA, USA) mounted directly above the arena. At a later time, videos were analyzed by one trained observer. Open field test (OFT) measures included latency to vocalize, number of vocalizations and defecations and time spent immobile in addition to binary assessment of whether a bird attempted to escape. An escape attempt was defined as two legs leaving the ground and jumping into, onto or over the test arena wall. 

### 2.3. Plasma Corticosterone Concentration 

After the OFT, blood samples were taken to measure the corticosterone response to the acute stress of the OFT (i.e., novelty, social isolation and handling). Blood samples were collected after the OFT test within 23–123 s for group A and 30–135 s for group B. Chickens were held on their side with neck extended and approximately 2 mL of blood was collected from the jugular vein with an S-monovette (Sarstedt AG & Co., Nümbrecht, Germany). After collection, blood samples were spun on site at 10,000 rpm for five minutes. The supernatant was collected, stored on ice and frozen at −20 °C for later analysis. Plasma corticosterone concentrations were measured using a commercially available double antibody radioimmunoassay kit (ImmuChem^TM^ Double Antibody Corticosterone 125I RIA kit, MP diagnostics, Orangeburg, NY, USA) as per manufacturer’s instructions with the exception of a 1:4 dilution factor to optimize detection within the standard curve. Duplicates with a coefficient of variation greater than 5% were reanalyzed. 

### 2.4. Novel Object Test

To assess neophobia and inspective curiosity, birds were subjected to a novel object test (NOT) in the same area as the OFT but the following day and in pairs. As such, the arena was not novel and the impact of social isolation was removed during the NOT. Birds were placed in the center of the bottom third of the arena, 1 m away from a novel object and left for five minutes. The novel object (NO) was a small florescent orange traffic cone (130 mm H × 70 mm W × 70 mm L). Behavior was recorded via a GoPro camera (Hero3, GoPro Inc., San Mateo, CA, USA) mounted above the arena and later assessed by one trained assessor blind to treatment. Time to reach the NO and binary score of ‘interacted with NO or not’ was recorded. The first bird out of the pair to approach and/or interact with the NO was recorded. The average distance of both chickens to the NO was measured via screenshots every 30 s and calculated using ImageJ software (Bethesda, MD, USA).

After the NOT, each chicken was weighed and leg health was assessed using a 5-point gait scoring method developed by Kestin et al. [23]. Leg health was expected to improve in the P group if perch use was frequent relative to C and HC birds that did not receive access to perches. 

### 2.5. Human Approach and Human Avoidance Tests

On day 38, birds were tested in the same arena as the OFT and NO and were tested in pairs for the human approach (HAP) and human avoidance (HAV) tests. Humans in the HAV and HAP were unfamiliar to the chickens (i.e., they were not the same human that applied the daily treatment) however all humans wore white laboratory coats during daily treatment and HAV/HAP tests. Birds were placed into the test arena and the human left the arena and testing room. After 2 min, the human entered the test arena and the HAP began. The human stood stationary in the middle of the area for 2 min with their hands by their side and looking forward (i.e., no eye contact with the chickens). Thereafter, the human walked to the corner of the arena furthest from the chickens and waited for 30 s with their back turned to the chickens and hands by their side. The human then turned to face the chickens and the HAV began; without making eye contact with the chickens the human walked towards the closest chicken at a pace of 1 step per second and its flight distance (i.e., the closest proximity from human before it moved away) was assessed. ‘Moving away’ was defined as taking two steps, or jumping (with or without wing flapping), in the direction away from the human. Tactile interactions with the human were also recorded. Behavioral responses were captured via a GoPro camera (Hero3, GoPro Inc., San Mateo, CA, USA) located above the arena and later assessed for distance from the human at 30 s intervals during the HAP and the flight distance of the focal chicken during the HAV. Distances were calculated using ImageJ (Bethesda, MD, USA) and were assessed by one assessor who was blind to treatment. 

### 2.6. Behavioral Time Budgets and Interaction with Environmental Enrichment

Behavioral time budgets and interaction with environmental enrichment were monitored hourly three times each week between 7:00–20:00 h by instantaneous scan sampling at one-hour intervals (Table 1). Days where birds were assessed for fearfulness (days 21–23 and 35–38) were excluded from the behavioral observations and two daily time points (8:00 h and 14:00 h) were excluded because a human was in the HC pens at those times to apply additional human contact treatment. Tactile interactions with human that were initiated by the birds (i.e., pecking and sitting on foot) were recorded during the HC treatment twice daily. All behavioral observations were made by one trained assessor via video recordings.

### 2.7. Statistical Analysis

All statistical analysis was performed with the SPSS statistical software (v22, IBM Corp., Armonk, NY, USA). Normality of data was assessed by Kolmogorov–Smirnov and Shapiro–Wilk normality test statistics and histograms. Latency to vocalize during the OFT data were not normally distributed and were square root transformed data; subsequently the transformed data met the criteria for normality and were used for analysis.

Corticosterone concentration, TI duration, flight distance during the HAV, number of tactile interactions with the human during the HC treatment and the total number of vocalizations, latency to vocalize, time spent frozen and number of defecations during the OFT were analyzed with general linear models. Ordinal data (number of TI inductions and gait scores) were analyzed with multinomial logistic regressions. Distance to NO during the NOT and the human during the HAP test were assessed using a repeated measure generalized linear mixed model with autoregressive correlation. Binary logistic models were utilized to determine the effect of treatment on whether a bird was still in TI at the end of the test, if a bird attempted to escape during the OFT and counts of tactile interactions with specific environmental enrichments items (total daily interaction with environmental enrichment items for each pen was the denominator). Behavioral time budgets were not normally distributed and were analyzed with a generalized linear mixed model with a Poisson distribution and log link function, but are presented as proportion of time (%) raw means. Models used to analyze fear responses were run for each age (21 vs. 35 days) independently. The experimental unit was considered to be the individual chicken in tests where they were tested individually, the pair of chickens for tests in pairs and the pen for in situ observations. Models that analyzed behavioral time budgets and interaction with enrichment included age (week) in all models and the interaction between age and treatment. All models included treatment as a fixed factor and room/pen as a random factor. Sex and the interaction between sex and treatment were included in models that assessed individual behavior, body weight, gait score and corticosterone concentrations. Effects were considered significant at *p* < 0.05 and considered a trend at *p* < 0.10.

## 3. Results

### 3.1. Interaction with the Environment and Environmental Enrichment

**Figure 2:**
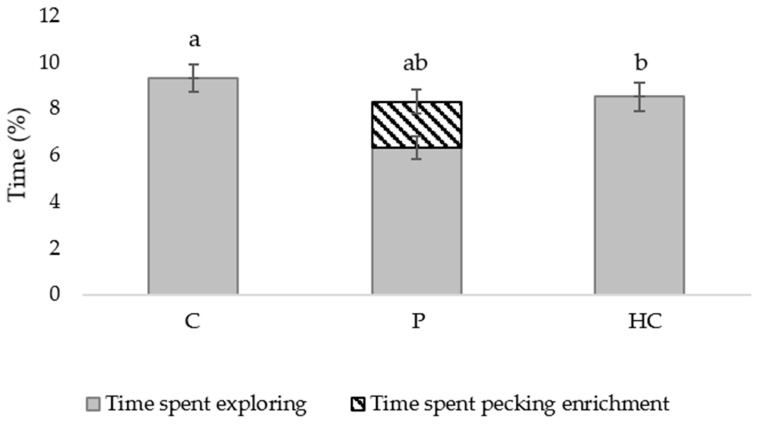
Mean proportion of time (±SEM) spent exploring (foraging and ground pecking; solid grey bars) and pecking at environmental enrichment items (striped bar; only applicable to P birds) between birds that were raised under standard conditions (C), with additional physical items (P) or with additional human contact (HC). Subscript (a, b, ab) refers to treatment differences when pecking enrichment was included to exploring; differing subscript denotes a treatment effect at *p* < 0.05. When pecking enrichment items was not included in the analysis (i.e., solid grey bars only) P birds spent less time exploring than C and HC birds (*p* < 0.001; subscript/significance not shown).

Chickens spent the most time exploring (foraging and ground pecking) during the second week of life (χ^2^_(4)_ 91.4, *p* < 0.001) and there was an overall effect of treatment (χ^2^_(2)_ 26.6, *p* < 0.001; Figure 2*)* but there was no interaction between age and treatment (*p* = 0.113). Overall, P birds spent less time exploring than both C (*p* = 0.005) and HC (*p* = 0.005) birds and C birds spent more time exploring than HC (*p* = 0.006) birds. However, when time spent pecking at environmental enrichment items was added to exploring there was no longer a difference between C and P birds (*p* = 0.123; Figure 2).

### 3.2. Behaivoral Time Budgets

**Figure 3:**
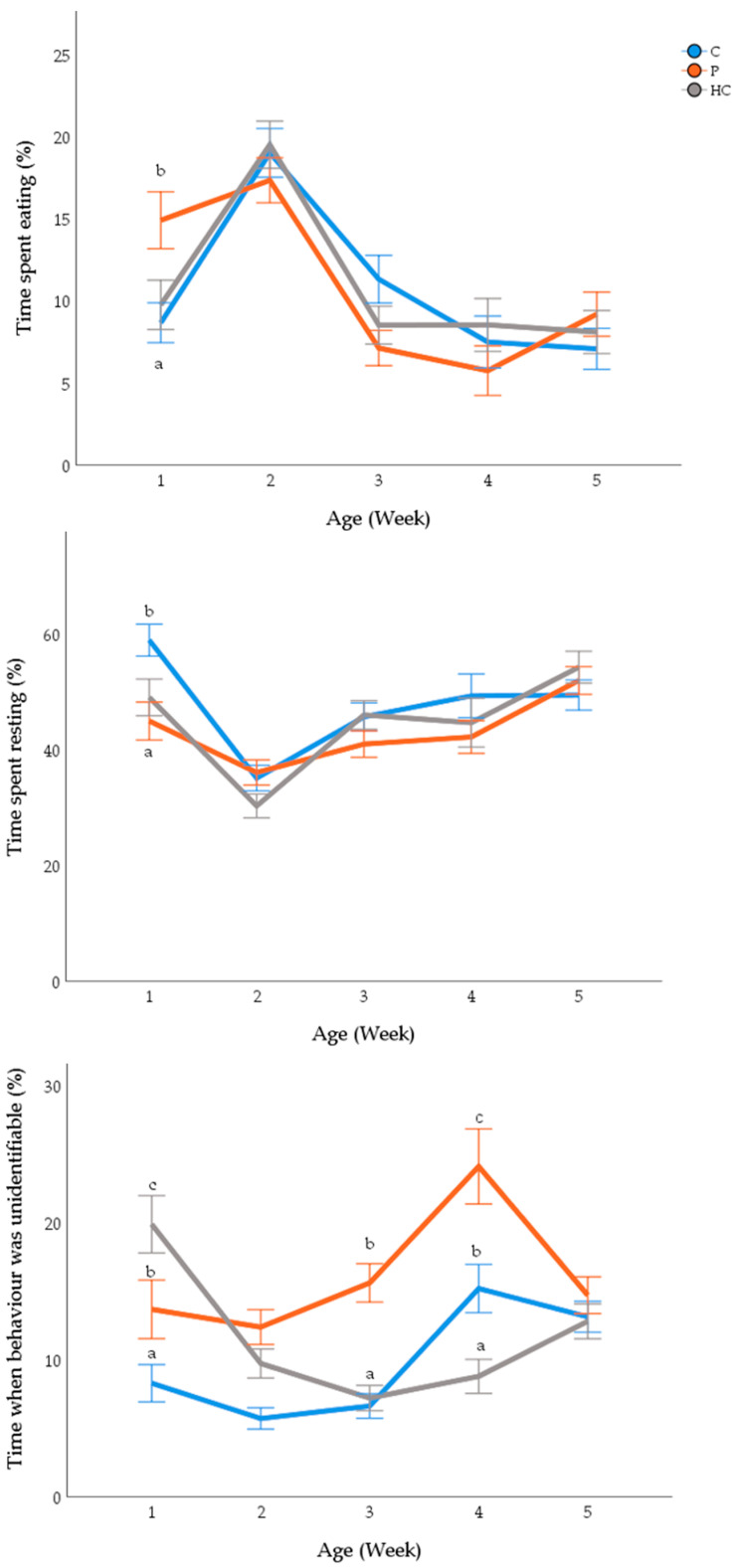
Mean proportion of time (±SEM) spent eating and resting and proportion of time that the behavior was unidentifiable from the 1st week of life to the last for birds that were raised under standard conditions (C, blue line), with additional physical items (P, grey line) or with additional human contact (HC, orange line). Differing subscript (a, b, c) denotes a treatment effect at *p* < 0.05.

There was an interaction between age and treatment for time spent resting (χ^2^_(8)_ 46.1, *p* < 0.001; Figure 3). C birds spent more time resting during the first week of life compared to P (*p* < 0.001) and HC (*p* < 0.001) birds. There was an interaction between age and treatment for time spent eating (χ^2^_(8)_ 32.1, *p* < 0.001; Figure 3) such that P birds spent more time eating during the first week of life compared to HC (*p* < 0.001) and C birds (*p* < 0.001). The time when behaviors could not be determined (‘’unidentifiable) differed with age and treatment (χ^2^_(8)_ 77.1, *p* < 0.001; Figure 3). There were more unidentifiable behaviors for the HC group during week 1 compared to C (*p* < 0.001) and P (*p* = 0.012), and more unidentifiable behaviors during weeks three and four for P birds compared to C (week 3, *p* = 0.014; week 4, *p* = 0.029) and HC (week 3, *p* = 0.014; week 4, *p* = 0.027).

Chickens spent more time performing comfort behaviors with increasing age (χ^2^_(4)_ 102, *p* < 0.001) and there was an effect of treatment (χ^2^_(2)_ 10.8, *p* = 0.005). Overall, P birds spent less time performing comfort behaviors than C (*p* = 0.003) and HC (*p* = 0.005) birds, of note whilst this was statistically different it only equated to an average of 1% less time performing comfort behaviors (Table 2 and Table S1). Chickens spent less time standing and walking with age (*p* < 0.001) but this was not related to treatment (standing: *p* = 0.124; walking: *p* = 0.462; Table 2 and Table S1). There was no effect of treatment on the expression of ‘other’ behaviors (i.e., play, interacting with conspecific and vigilance) (*p* = 0.376) and there was no interaction between age and treatment (*p* = 0.384). There was no effect of treatment, age or their interaction on drinking behavior (*p* > 0.05; Table 2 and Table S1).

### 3.3. Interacting with Physical Environmental Enrichment Items

**Figure 4:**
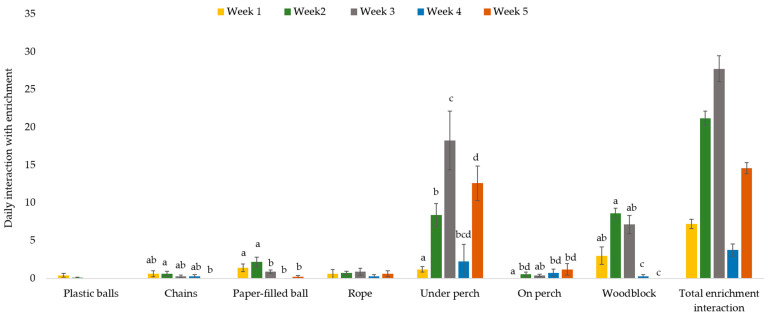
Average daily counts of interactions with environmental enrichments observed during hourly scan samples between 7:00–20:00 for birds provided with physical items (P birds only). Differing subscript (a, b, c, d) indicates significant difference of time spent interacting with a specific environmental enrichment over time/age (*p* < 0.05).

Interaction with the environmental enrichment items (P birds only) as a whole did not change over time (*p* = 0.929). However, use of specific environmental enrichments changed over time (age × environmental enrichment interaction (χ^2^_(17)_ = 73.3, *p* < 0.0001). Of the pecking objects (plastic balls, chains, rope and paper-filled ball), the most preferred was the paper-filled ball (all pairwise comparisons *p* < 0.05), although interest in the paper-filled ball significantly decreased from week three onward (Figure 4) despite the colored paper being regularly replenished. Overall, there were few tactile interactions with the small plastic colored balls or rope and there was no change with age (all pairwise comparisons *p* > 0.05; Figure 4). Tactile interactions with chains decreased from weeks two to five but interaction at all time points was low (all pairwise comparisons *p* > 0.05; Figure 4). Overall, chickens interacted the least with the small plastic balls (all pairwise comparisons *p* < 0.05) and the most with the perch (specifically sitting under it) and the woodblock (all pairwise comparisons *p* < 0.05). Use of the woodblock decreased with age, which was likely associated with increased body size which became too large relative to the dimensions of the woodblock. Chickens were more often found sitting underneath the perch rather than perching on it (*p* < 0.05; Figure 4).

### 3.4. Interacting with Human during Human Contact Treatments

**Figure 5:**
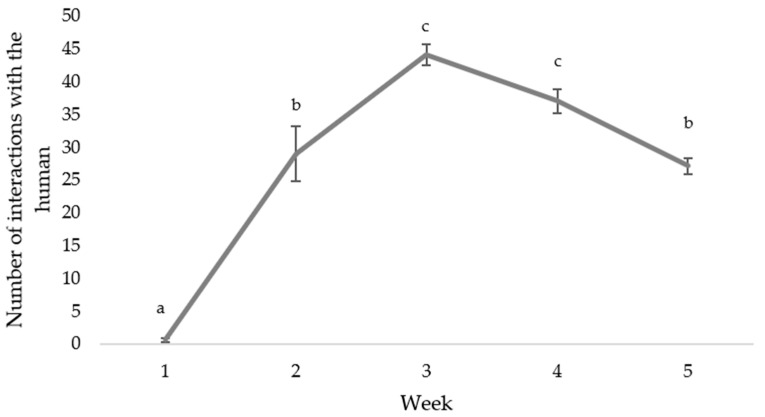
The average number of tactile interactions during the stationary phase of a 5-min Human Contact (HC) treatment from the first to last week of life. An interaction was defined as any tactile interaction with the human (e.g., pecking the human or sitting on the human’s foot) and was always initiated by the chicken with no behavioral response or attempt to interact from the human at any time. Differing subscript (a, b, c) indicates a significant difference at *p* < 0.05.

Tactile interactions with the human during the HC daily treatment showed that there was no difference between interaction with the human in the morning and the afternoon sessions (*p* = 0.774), and therefore counts were averaged as a daily human interaction score for each pen. Comparisons between weeks indicated that interaction with the human was the lowest during week one, increased during week two, peaked during week three and decreased thereafter (F_(4,73)_ = 36.5, *p* < 0.001; Figure 5).

### 3.5. Fearfulness

At 21 days of age there was an effect of treatment on the number of inductions required to induce a state of TI (χ^2^_(2)_ = 6.83, *p* = 0.03; Table 3) but not at 35 days of age (*p* = 0.217). At 21 days of age, the P chickens required more inductions to enter a state of TI than C chickens (*p* = 0.02) and HC chickens (*p* = 0.02). However, TI duration (21 days: *p* = 0.646; 35 days: *p* = 0.584) or whether a chicken was still in TI after 10 min (21 days: *p* = 0.666; day 35: *p* = 0.940; Table 3) did not differ between treatments (Table 3).

There was no difference between treatments in any of the indicators of fearfulness assessed during the open field test (all *p* > 0.05; Table 4 and Table S2). 

Males were more likely to vocalize during the OFT than females at both 21 days of age (F_(1,41)_ = 9.01, *p* = 0.005) and 35 days of age (F_(1,45)_ = 4.34, *p* = 0.043), but there was no interaction between sex and treatment (*p* > 0.05; Table 4 and Table S2).

There was no effect of treatment on plasma corticosterone concentrations after the OFT at 21 days (C: 4.1 ± 0.4 ng/mL; P: 4.0 ± 0.5 ng/mL; HC: 4.4 ± 0.4 ng/mL; *p* = 0.997) or 35 days (C: 3.1 ± 0.4 ng/mL; P: 2.4 ± 0.3 ng/mL; 2.5 ± 0.3 ng/mL; *p* = 0.536). There was a significant effect of sex on corticosterone at 21 days (F_(1,31)_ = 9.3, *p* = 0.004) and a trend at 35 days of age (F_(1,39)_ = 3.7, *p* = 0.061) but there was no interaction between treatment and sex (21 days *p* = 0.234; 35 days *p* = 0.389).

**Figure 6:**
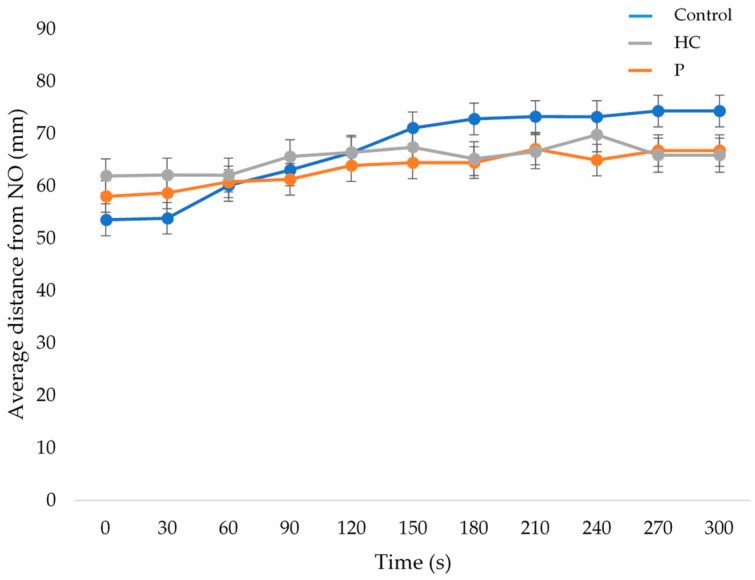
Average distance (±SEM) from a novel object (traffic cone) of a pair of birds every 30 s after they were placed in a test arena at 22 days of age. Birds were raised under standard conditions (control, blue line); with additional physical items (P, grey line); or with additional human contact (HC, orange line). Results are presented for birds that were tested at 21 days of age.

No bird at either age from any treatment interacted with the novel object. There was a time by treatment interaction for distance from the NO at 21 days of age (F_(20,188)_ = 2.4, *p* = 0.001) but not at 35 days of age (*p* = 0.261). At 21 days of age, C birds moved away from the NO overtime but the HC and P birds did not (Figure 6). Birds moved away from the NO overtime at 35 days of age (F_(10,237)_ = 3.9, *p* < 0.001), but there was no effect of treatment (*p* = 0.558).

There was an effect of treatment on the HAP (F_(2,39)_ = 3.20, *p* = 0.052) and HAV (F_(2,42)_ = 4.34, *p* = 0.019). Chickens from the HC group were closer to the human during the human approach compared to the C (*p* = 0.034) and P (*p* = 0.04) chickens. HC chickens had a shorter flight distance when approached during the HAV than C chickens (*p* = 0.006) but not P chickens (*p* = 0.368); P chickens tended to have a shorter flight distance than C chickens (*p* = 0.084; Table 5 and Table S3).

### 3.6. Body Weight

There was no difference in body weight between treatments at 21 days of age (C: 864.2 ± 31.3g; P: 860.7 ± 22.6g; HC: 818.5 ± 23.5g; *p* = 0.715) or 35 days of age (C: 2161.8 ± 54.0g; P: 2064.9 ± 69.3g; HC: 2140.0 ± 67.9g; *p* = 0.186). Males tended to be heavier than females at 21 days of age (F_(1,42)_ = 2.86, *p* = 0.098) and males were heavier than females at 35 days of age (F_(1,46)_ = 31.5, *p* < 0.001). However, there was no interaction between sex and treatment at either age (21 days of age *p* = 0.339; 35 days of age *p* = 0.357).

### 3.7. Leg Health

There was no effect of treatment on gait score at 21 days of age (*p* = 0.692) or 35 days (*p* = 0.832; Table 6). There was no main effect of sex on gait score nor an interaction between sex and treatment at either age (*p* > 0.05).

## 4. Discussion

While we found little evidence that providing additional environmental complexity altered general fearfulness, stress physiology, body weight or leg health of meat chickens, the addition of daily positive human contact reduced fear of humans. Although chickens from the physical item treatment group interacted with the additional physical items in their home pens, this pecking and exploratory behavior was directed towards the litter in the other two treatment groups. Interaction with chains, ropes and small plastic balls was very low and reduced over time. Chickens preferred to sit under the perch than on top of it across all five weeks of life. We provide evidence that these physical ‘environmental enrichment’ items unlikely ‘enriched’ the meat chicken’s environment in a way that was sustained over time or resulted in welfare benefits based on the measures we collected.

We aimed to increase agency in our chickens that were provided with additional physical items, which we predicted would result in more interactions with the environment and willingness to approach the novel object (small florescent orange cone) in the novel object test. Yet we found little evidence of this effect. We do show that a chicken’s motivation to peck is very strong. Even when chickens did not have additional pecking devices in their pens, pecking behavior was directed to the litter. Strings and chains are often provided on Australian meat chicken farms as a form of environmental enrichment (personal communication), providing resources to appease the chickens’ desire to peck [24]. However, the low use of chains reported in this study and the reduction over time suggests that these devices are not rewarding enough to reinforce use, in agreement with Bailie and O’Connell [25] and Arnould et al. [26]. Providing more biologically relevant physical environmental enrichments may have increased time spent pecking. Indeed, the paper-filled ball was the most preferred pecking item, which was likely the most biologically relevant; such that it may have mimicked foraging in grass, straw and/or haybales. However, the preference for the paper-filled ball may also reflect the dynamic nature of the environmental enrichment, such that it could be manipulated and moved causing change in the texture and appearance. The importance of novelty to maintain positive interactions with their environment could also be seen in the human contact group, as interaction with the human increased as fear of humans presumably decreased, peaking at week three and then reduced, suggesting that the chickens no longer found the interaction rewarding. 

There was very little use of the perch to sit on by the chickens in the environmental complexity group. Despite various welfare quality assurance programs mandating the provision of perches (for example RSPCA Australia [24]) research regularly reports low use of perches by meat chickens (8% of birds Estevez et al. [27]; 10% of birds Matkovic et al. [28]) in agreement with our findings. However, to truly quantify perch use, individuals should be identified when assessing use of the perch as scan sampling can underestimate the use of such a resource because use may occur in a cyclical fashion (i.e., chickens may not use the resource at the same time) as observed with monitoring flock vs. individual range use [29]. However, it may be that our perch design was not attractive to meat chickens, which may have been related to the height of the perches. Meat chickens have been shown to prefer higher perches (15 cm) compared to lower (7 cm) [27]. Our results suggest that 20cm perches may be too high for meat chickens or that meat chickens prefer very low perches (2.5 cm) as we found that chickens preferred the woodblock (2.5 cm) compared to the perch (20 cm). However, the space available on the woodblock was limited (one chicken maximum at any age) which may have altered use. We provided wide perches (70 mm) that were closer to platforms than traditional perches which aligns with broiler preference [27,30] but may have not been wide enough, or perhaps required ramps to encourage use. The finding that chickens preferred to sit underneath the perch rather than on top of it warrants consideration. Very few studies have investigated the importance of cover inside poultry sheds, in contrast to outdoor shelter provision. However, the scientific findings are clear that chickens prefer to inhabit areas with cover not only in outdoor areas [31,32] but also inside a shed [33]. This is not surprising considering the ancestral environments of dense jungle habitat [34] and the level of cover provided by mother hens during the first few weeks of life [35]. Proving overhead structures (which may also serve as perching structures as in the current study) should be considered. Of note, use of the space under the perches increased with time/age and may have been an indirect effect of reduced space (increased stocking density) rather than a preference to utilize this space. Although, the preference was observed during the second week of life when space would have been ample we cannot rule out the impact of increased stocking density on utilization of space. 

The effect of environmental complexity on meat chicken behavioral time budgets warrants further investigation, as the significant relationship between age and treatment on unidentifiable behaviors in the current study may have masked treatment effects, or resulted in false positive treatment effects. More birds from the physical item treatment group were unable to be seen or their behavior identified with increasing age. The increased amount of unidentifiable location or behavior of P chickens correlates with time spent under the perch which may explain the unidentifiable behavior, i.e., the observer could see they were under the perch but could not tell what behavior they were performing. Therefore, the observed treatment effects (i.e., less comfort behavior observed for P birds compared to C and HC birds) should be considered carefully, as P birds may have preferred to express comfort behaviors when located under the perch. However, more time spent eating and less time resting when birds were provided with additional physical items agrees with the literature that shows some environmental enrichment can increase activity of meat chickens [25,36,37]. Yet, the least amount of unidentifiable behaviors observed for C compared to P and HC during the first week of life were not expected and cannot be easily explained. 

The duration of TI did not differ between treatment groups, however chickens that received increased environmental complexity required more attempts to induce TI than chickens from the other treatments, providing some evidence that environmental complexity by providing chickens with physical items may reduce fearfulness at 21 days of age. However, the evidence was not strong, nor was it sustained until 35 days of age. Susceptibility to TI (i.e., the number of inductions required to induce TI), has shown to increase after chickens are administered an anxiogenic drug [38] or poor handling [39]. However, typically this effect is associated with an increase in the duration of the TI state, unlike the disparity observed in the current study. The small sample size in the current study may have overestimated the proportion of birds that required more inductions or was not sufficient to show statistical differences between the environmental complexity and control groups; the duration of TI was on average 10s shorter for birds provided with physical items. These results cannot therefore conclusively suggest that increased environmental complexity does not impact fearfulness at 21 days of age. The importance of assessing fear at 21 days of age is evident in the Australian free-range chicken meat industry, as access to the outdoor range is typically provided from 21 days of age when birds are sufficiently feathered to cope with various weather conditions. Stadig et al. [40] provided some evidence that fearfulness was related to range use in slow-growing chickens. As such, reducing fearfulness at 21 days of age could improve utilization of an outdoor range by fast-growing meat chickens. Although, Taylor et al. [41] found no relationships with fearfulness before range access (i.e., fearfulness did not impede range use, but was reduced later in life if a chicken spent more time spent on the range), curiosity (which is thwarted by fearfulness) may be an important characteristic. The novel object test provided no evidence that a more complex environment increased curiosity, rather control chickens moved further away from the novel object at 21 days suggesting fearfulness (but not curiosity) was affected by treatment. A broader benefit for reduced fearfulness would be a reduction of fearfulness at 35 days of age when in Australia one third of fast growing meat chicken flocks are typically picked up for transportation and slaughter. Improving stress resilience to such events would likely be beneficial for chicken welfare and production (for example, fewer downgrades from injuries caused from fear and panic [42]). Zulkifli and Siegel [10] argue that increased novelty in an environment improves stress resilience, rather than simply environmental complexity. Although the environment provided to the physical items group in the current study was more complex than typical rearing environments, the enrichment items were not dynamic which may be the key to reducing fearfulness and increasing curiosity in meat chickens. Indeed, Altan et al. [43] were able to reduce meat chicken fearfulness, evident by shorter duration of tonic immobility, by providing toys that were not biologically relevant (for example, plastic balls, plastic bottles and mirrors) but were changed every three days. Although seemingly effective, providing novelty in an environment by changing physical items every few days is impractical to implement in commercial conditions, but other forms of novelty that are more practical to implement (i.e., access to a novel area in the shed [44] or outdoors [36] or intermittent laser lights [43]) could be further investigated. 

We showed no difference between treatment groups in their physiological stress responses. However, our results may reflect basal (or close to) corticosterone plasma concentrations rather than the corticosterone response to the OFT as ACTH challenges in meat chickens show a peak of corticosterone concentration in plasma after 30 min [45]. Of note, shorter time frames than 30 min have successfully captured the physiological stress differences of meat chickens. For example, Hemsworth, Coleman, Barnett and Jones [20] showed differences in physiological stress responses of broilers that received positive handling compared to chickens that did not receive additional handling, after 12 min of being confined in a crate, well below the peak corticosterone response reported by Post, Rebel and ter Huurne [45] demonstrating that the variation in peak physiological stress responses are dependent on the nature (i.e., intensity and duration) of the stressor [46]. 

We were able to successfully reduce the fear responses of chickens to humans by providing chickens with 5 min of additional positive visual contact twice daily. This effect was not observed in measures of general fearfulness, which may be due to a stimulus-specific effect, or missing a critical sensitive period. Zulkifli, Gilbert, Liew and Ginsos [22] provided visual human contact to meat chickens for 10 min each day either for their whole life (0–6 weeks), the first three weeks of life or in the last three weeks of life (3–6 weeks of age). Birds that were exposed to humans for the whole of their lives, or during the first three weeks, showed lower general fearfulness long term (evident by shorter TI durations), that was not observed in chickens that received additional human contact from three to six weeks of life. Our human contact treatment was only implemented on the fifth day of life and did not yield similar results to Zulkifli, Gilbert, Liew and Ginsos [22]. Together, this may be evidence that the first three days of life are important to target any interventions that utilize human contact to reduce general fearfulness. Of note, the type of interaction with humans may also be important. The current study and that by Zulkifli, Gilbert, Liew and Ginsos [22] report the impacts of early life exposure to visual contact with humans, however Jones [21] found no evidence of a sensitive period to reduce fear of humans when tactile methods (regular positive handling) were applied. Nonetheless, a reduction in fear of humans can have important implications for both animal welfare and stockperson attitudes (see Hemsworth and Coleman [47] and Zulkifli [48]). Positive handling with increased visual human contact has shown to improve immune function [22,49] and production [50] in poultry. Daily handling of poultry by humans is clearly impractical for large commercial flocks but the positive impact of increased visual human contact should be further investigated in large scale commercial conditions as the aforementioned evidence from small scale research trials, including the results from the current study, are promising.

## 5. Conclusions

We found relatively little evidence that increasing environmental complexity by providing physical items altered the chickens’ behavioral time budget, fear, physiological stress response, leg health or production measures. Furthermore, the physical items were not sustainably valued by chickens as evidenced by little, and reduced, use over time. Additional human visual contact reduced fear of humans but did not alter general fear responses. The sensitive period for this effect should be further investigated, as well as how this could be applied in commercial settings in larger flocks. Low utilization of the physical enrichment items indicate that these items are not suitable ‘enrichment’ items for on farm use and further investigation is required to identify physical items that are rewarding and improve welfare. 

## Figures and Tables

**Table 1:** Ethogram to assess the behavioral time budgets of meat chickens.

Category	Behavior	Description
Exploring	Foraging	Pecking at the ground whilst scratching, kicking or digging in the substrate
	Ground pecking	Pecking at the ground whilst walking, standing or sitting
Interacting with enrichment	Interacting with enrichment	Tactical interaction with any physical enrichment item
Resting	Resting	Breast and hocks touching the ground underneath the body or on either side of the body. Head is positioned either under wing or upright, the neck is not extended, there is no movement
Walking	Walking	Stepping legs to initiate movement, there is no movement of the wings. At least two steps in a forward direction
Eating	Eating	Head over the feeder moving up and down in a pecking motion
Drinking	Drinking	Head over the lip of the bell drinker with intermittent periods with head raised high and extended.
Comfort	Preening	Head turned into body with movement, head may extend away returning to previous posture within 10 s
	Wing flapping	Both wings are extended, approximately 90° from body and vigorously shaken up and down. This behavior is performed standing or whilst running
	Dustbathing	Bird in contact with substrate, shaking wings up and down rigorously intermittently with lying still position
Other	Play	Running in a non-linear direction whilst flapping wings. The behavior is not directed at a conspecific
	Interaction with conspecific	Gentle successive contact with beak on a conspecific.
	Vigilance	Neck extended away from body, head raised and alert, bird is either frozen or there is continuous rotation of the head, in either a sitting or standing position
	Aggression	Threat: Neck extended above a conspecific, conspecific head is down low to the ground (submissive behavior), feathers ruffled and bird is standing. Aggressive peck: Beak in successive contact with force to a conspecific.
Unidentifiable		Behavior cannot be determined and/or bird cannot be seen in camera view

**Table 2:** Mean proportion of time (±SEM) spent drinking, standing, walking and performing comfort or other behaviors for birds that were raised under standard conditions (C); with physical items (P); or with additional human contact (HC). Differing superscript indicates a significant difference at *p* < 0.05 between treatment groups. Bold font indicates significant *p*-values at *p* < 0.05.

	C	P	HC		*p* Value	
				Treatment	Age	Treatment × Age
Drinking (%)	2.6 ± 0.3	2.8 ± 0.3	2.8 ± 0.3	0.728	0.670	0.722
Standing (%)	7.1 ± 0.6	7.2 ± 0.6	8.3 ± 0.6	0.124	**<0.001**	0.066
Walking (%)	5.9 ± 0.4	6.0 ± 0.5	6.5 ± 0.5	0.462	**<0.001**	0.104
Comfort (%)	7.4 ± 0.5 ^a^	6.0 ± 0.4 ^b^	7.7 ± 0.5 ^a^	**0.005**	**<0.001**	0.163
Other (%)	1.0 ± 0.2	0.8 ± 0.2	0.9 ± 0.2	0.376	**0.001**	0.384

**Table 3:** Number of inductions required to induce a state of tonic immobility (maximum 5), duration of tonic immobility (TI) and the proportion of chickens that stayed in TI for the maximum time permitted (10 min) for chickens that were raised under standard conditions (C); with additional physical items (P); or with additional human contact (HC). Differing superscript within a column indicates treatment differences at *p* < 0.05.

Age	Trt	Number of Inductions Required to Induce TI					Duration of TI (s)	Max time in TI (%)
		1 (%)	2 (%)	3 (%)	4 (%)	5 (%)		
21	C	55.6 ^a^	33.3 ^a^	11.1	0.0 ^a^	0.0 ^a^	200 ± 30	16.7
	P	22.2 ^b^	50.0 ^b^	11.1	5.6 ^b^	11.1 ^b^	191 ± 35	25.0
	HC	52.9 ^a^	35.3 ^a^	5.9	5.9 ^b^	0.0 ^a^	204 ± 31	29.4
35	C	29.4	35.3	5.9	11.8	17.6	212 ± 37	40.0
	P	61.1	30.8	5.6	0.0	11.1	186 ± 24	35.3
	HC	55.6	16.7	0.0	0.0	27.8	193.6 ± 20.0	28.6

**Table 4:** Indicators of fearfulness (mean ± SEM or proportion of treatment group) during the open field test for chickens that were raised under standard conditions (Control; C); with additional physical items (P); or with additional human contact (HC). Bold font indicates significant *p*-values at *p* < 0.05.

Age	Indicator	C	P	HC	*p*-Value		
					Treatment	Sex	Trt × Sex
21 days	Vocalizations	207.9 ± 18.5	245.2 ± 19.1	228.1 ± 18.3	0.389	**0.005**	0.138
	Latency to vocalize (s)	12.9 ± 2.8	6.3 ± 2.9	6.6 ± 2.9	0.174	0.331	0.769
	Time spent immobile (s)	6.1± 3.6	6.4 ± 3.9	4.4 ± 3.8	0.949	0.704	0.903
	Attempted to escape (%)	33.3	16.7	50.0	0.233	0.254	0.569
	Defecations	1.4 ± 0.2	1.1 ± 0.2	1.4 ± 0.2	0.523	0.087	0.841
35 days	Vocalizations	112.0 ± 16.0	113.8 ± 14.7	97.2 ± 14.8	0.710	**0.043**	0.995
	Latency to vocalize (s)	9.97 ± 3.7	13.2 ± 3.5	14.8 ± 3.5	0.589	0.064	0.562
	Time spent immobile (s)	9.9 ± 4.0	5.2 ± 3.8	9.0 ± 3.8	0.844	0.271	0.366
	Attempted to escape (%)	17.6	16.7	22.2	0.411	0.297	0.174
	Defecations	1.1 ± 0.2	1.4 ± 0.2	1.0 ± 0.6	0.414	0.604	0.643

**Table 5:** Mean (±SEM) distance from stationary human (HAP) and the distance the chicken moved away from the approaching human (HAV) when chickens that were raised under standard conditions (Control); with additional physical items (P); or with additional human contact (HC). Differing superscript within a column indicates treatment differences at *p* < 0.05. Bold font indicates significant *p*-values at *p* < 0.05.

	C	P	HC	*p* Value
HAP (cm)	66.8 ± 4.5 ^a^	66.7 ± 4.8 ^a^	53.5 ± 4.1 ^b^	**0.052**
HAV (cm)	38.4 ± 5.0 ^a^	25.1 ± 5.7 ^a^	18.4 ± 4.7 ^b^	**0.019**

**Table 6:** Gait scores of chickens at 21 and 35 days of age. Chickens were raised under standard conditions (Control; C), with physical items (P) or with additional human contact (HC).

Age	Trt	GS0 (%)	GS1 (%)	GS2 (%)	GS3 (%)
21	C	37.5 (*n* = 6)	25.0 (*n* = 5)	37.5 (*n* = 6)	0.0 (*n* = 0)
	P	33.3 (*n* = 6)	27.8 (*n* = 4)	33.3 (*n* = 6)	5.6 (*n* = 1)
	HC	50.0 (*n* = 8)	6.3 (*n* = 1)	37.5 (*n* = 6)	6.3 (*n* = 1)
35	C	0.0 (*n* = 0)	33.3 (*n* = 5)	60.0 (*n* = 9)	6.7 (*n* = 1)
	P	5.6 (*n* = 1)	50.0 (*n* = 9)	38.9 (*n* = 7)	5.6 (*n* = 1)
	HC	5.6 (*n* = 1)	44.4 (*n* = 8)	38.9 (*n* = 7)	11.1 (*n* = 2)

## Data Availability

Data is available at https://doi.org/10.6084/m9.figshare.19067966.

## Supplementary Materials

The following supporting information can be downloaded at: https://www.mdpi.com/article/10.3390/ani12030310/s1, Table S1: Estimated marginal means of the proportion of time spent drinking, standing, walking and performing comfort or other behaviors for birds that were raised under standard conditions (C); with physical items (P); or with additional human contact (HC).; Table S2: Indicators of fearfulness (estimated marginal means and SEM) during the open field test for chickens that were raised under standard conditions (Control; C), with additional physical items (P) or with additional human contact (HC) and model SEM are reported for birds tested at 21 and 35 days of age; Table S3: Estimated marginal means distance from stationary human (HAP) and the distance the chicken moved away from the approaching human (HAV) when chickens that were raised under standard conditions (Control); with additional physical items (P); or with additional human contact (HC).
