# Supplementary material for: Environmental Complexity: Additional Human Visual Contact Reduced Meat Chickens’ Fear of Humans and Physical Items Altered Pecking Behavior
Source: Animals (Basel). 2022 Jan 27;12(3):310. doi: 10.3390/ani12030310 (PMC8833824; doi:10.3390/ani12030310)
Supplement: Supplementary file 1 [file animals-12-00310-s001.zip › animals-1532676-SI.pdf]

Table S1. Estimated marginal means of the proportion of time spent drinking, standing, walking and performing comfort or other behaviors for birds that were raised under standard conditions (C); with physical items (P); or with additional human contact (HC). Differing superscript indicates a significant difference at $p < 0.05$ between treatment groups. Bold font indicates significant p-values at $p < 0.05$.

	C	P	HC	SEM	*p*-Value		
					Treatment	Age	Treatment × Age
Drinking	0.27	0.29	0.29	0.02	0.728	0.670	0.722
Standing	0.73	0.72	0.85	0.04	0.124	**<0.001**	0.066
Walking	0.58	0.57	0.64	0.03	0.462	**<0.001**	0.104
Comfort	0.72^{a}	0.54^{b}	0.72^{a}	0.03	**0.005**	**<0.001**	0.163
Other	0.00	0.00	0.00	0.01	0.376	**0.001**	0.384

Table S2. Indicators of fearfulness (estimated marginal means and SEM) during the open field test for chickens that were raised under standard conditions (Control; C), with additional physical items (P) or with additional human contact (HC) and model SEM are reported for birds tested at 21 and 35 days of age. Bold font indicates significant p-values at $p < 0.05$.

Age	Variable	C	P	HC	SEM	*p*-Value		
						Treatment	Sex	Trt × Sex
21 days	Vocalizations	205.7	242.8	240.0	10.7	0.389	**0.005**	0.138
	Latency to vocalize (s)	13.1	6.5	6.7	1.42	0.174	0.331	0.769
	Time spent immobile (s)	5.7	6.2	4.5	1.94	0.949	0.704	0.903
	Attempted to escape (%)	0.8	0.8	0.6	0.07	0.233	0.254	0.569
	Defecations	1.3	1.0	1.4	0.12	0.523	0.087	0.841
35 days	Vocalizations	76.1	46.6	41.6	8.70	0.710	**0.043**	0.995
	Latency to vocalize (s)	10.4	13.5	13.5	1.90	0.589	0.064	0.562
	Time spent immobile (s)	11.3	5.6	8.3	2.27	0.844	0.271	0.366
	Attempted to escape (%)	0.8	1.0	0.8	0.05	0.411	0.297	0.174
	Defecations	1.02	1.34	1.07	0.13	0.414	0.604	0.643

Table S3. Estimated marginal means distance from stationary human (HAP) and the distance the chicken moved away from the approaching human (HAV) when chickens that were raised under standard conditions (Control); with additional physical items (P); or with additional human contact (HC). Differing superscript within a column indicates treatment differences at $p < 0.05$. Bold font indicates significant p-values at $p < 0.05$.

	C	P	HC	SEM	*p*-Value
HAP (cm)	65.5 ± 4.3^{a}	67.6 ± 4.3^{a}	53.1 ± 4.1^{b}	2.5	**0.052**
HAV (cm)	38.0 ± 4.9^{a}	29.5 ± 5.2^{a}	18.9 ± 4.7^{b}	3.1	**0.019**
